# CSN6‐SPOP‐HMGCS1 Axis Promotes Hepatocellular Carcinoma Progression via YAP1 Activation

**DOI:** 10.1002/advs.202306827

**Published:** 2024-02-02

**Authors:** Kai Li, Jiayu Zhang, Haiwen Lyu, Jinneng Yang, Wenxia Wei, Yuzhi Wang, Haidan Luo, Yijing Zhang, Xin Jiang, Hairong Yi, Mengan Wang, Caiyun Zhang, Kang Wu, Lishi Xiao, Weijie Wen, Hui Xu, Guolin Li, Yunle Wan, Fang Yang, Runxiang Yang, Xinhui Fu, Baifu Qin, Zhongguo Zhou, Haipeng Zhang, Mong‐Hong Lee

**Affiliations:** ^1^ Guangdong Provincial Key Laboratory of Colorectal and Pelvic Floor Disease The Sixth Affiliated Hospital Sun Yat‐sen University Guangzhou 510655 China; ^2^ Guangdong Research Institute of Gastroenterology The Sixth Affiliated Hospital Sun Yat‐sen University Guangzhou 510655 China; ^3^ Key Laboratory of Human Microbiome and Chronic Diseases (Sun Yat‐sen University) Ministry of Education Guangzhou 510655 China; ^4^ Biomedical Innovation Center The Sixth Affiliated Hospital Sun Yat‐sen University Guangzhou 510655 China; ^5^ Department of Experiment & Research South China Hospital Shenzhen University Shenzhen 518116 China; ^6^ Department of Hepatobiliary Pancreatic and Splenic surgery The Sixth Affiliated Hospital Sun Yat‐sen University Guangzhou 510655 China; ^7^ Department of the Second Medical Oncology The Third Affiliated Hospital of Kunming Medical University Kunming Yunnan Province 650118 China; ^8^ Collaborative Innovation Center for Cancer Medicine State Key Laboratory of Oncology in South China Sun Yat‐Sen University Cancer Center Guangzhou Guangdong P. R. China 510060; ^9^ Department of Pharmacology School of Medicine Jinan University Guangzhou 510632 China

**Keywords:** cholesterol metabolism, hepatocellular carcinoma(HCC), HFD, YAP1

## Abstract

Cholesterol metabolism has important roles in maintaining membrane integrity and countering the development of diseases such as obesity and cancers. Cancer cells sustain cholesterol biogenesis for their proliferation and microenvironment reprograming even when sterols are abundant. However, efficacy of targeting cholesterol metabolism for cancer treatment is always compromised. Here it is shown that CSN6 is elevated in HCC and is a positive regulator of hydroxymethylglutaryl‐CoA synthase 1 (HMGCS1) of mevalonate (MVA) pathway to promote tumorigenesis. Mechanistically, CSN6 antagonizes speckle‐type POZ protein (SPOP) ubiquitin ligase to stabilize HMGCS1, which in turn activates YAP1 to promote tumor growth. In orthotopic liver cancer models, targeting CSN6 and HMGCS1 hinders tumor growth in both normal and high fat diet. Significantly, HMGCS1 depletion improves YAP inhibitor efficacy in patient derived xenograft models. The results identify a CSN6‐HMGCS1‐YAP1 axis mediating tumor outgrowth in HCC and propose a therapeutic strategy of targeting non‐alcoholic fatty liver diseases‐ associated HCC.

## Graphical abstract

Cancer cells sustain cholesterol biogenesis for their proliferation and microenvironment reprograming even when sterols are abundant. However, efficacy of targeting cholesterol metabolism for cancer treatment is always compromised. The results identify a CSN6‐HMGCS1‐YAP1 axis mediating tumor outgrowth in hepatocellular carcinoma (HCC) and propose a therapeutic strategy of targeting non‐alcoholic fatty liver diseases‐associated HCC.

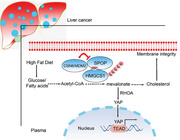

## 1 Introduction

Liver cancer, 90% of primary liver cancers are HCC, is increasing globally and is the second leading cause of cancer‐related death.^[^ ^1^ ^]^ The liver serves as a central metabolic coordinator with a wide array of essential functions, including the regulation of glucose, lipid metabolism and bile acid synthesis.^[^ ^2^ ^]^ The major risk factors of HCC include hepatitis viral infection, aflatoxin contamination and liver metabolic diseases. Among these risk factors, nonalcoholic fatty liver diseases (NAFLD) is predicted to become the leading cause of HCC in the future as a secondary consequence of the obesity pandemic.^[^ ^3^ ^]^ However, treatment of NAFLD‐associated cancer remains to be further explored.

Cholesterol has multiple physiological roles, including modulation of membrane function, involving protein posttranslational prenylation, serving as a precursor to bile acid, inducing chronic inflammation, and modifying tumor microenvironments.^[^ ^4^ ^]^ Cholesterol is synthesized from acetyl‐CoA through about 30 steps of mevalonate (MVA) pathway.^[^ ^5^ ^]^ Although high levels of cholesterol and other lipids are major contributing factors for NAFLD‐related cancer,^[^ ^6^ ^]^ the enzymes of MVA pathways involved in cancer have not been fully characterized. It has been shown that cholesterol in the tumor microenvironment can cause CD8^+^ T cell expression of immune checkpoints and subsequent exhaustion.^[^ ^7^ ^]^ The MVA pathway inhibitors (lipophilic statins, FPPS and GGPPS Inhibitors) can be employed for synergizing with anti‐PD‐1 antibodies.^[^ ^8^ ^]^ Despite these observations, identifying and validating mechanisms of MVA pathway dysregulation in liver cancer remain unmet challenges.

CSN6 (gene name, COPS6), a member of the COP9 protein complex that has previously been implicated in signal transduction and tumorigenesis, is a multi‐functional protein that regulates much of the protein turnover in eukaryotic cells through Cullin‐RING ubiquitin ligase (CRL) complex.^[^ ^9^ ^]^ CRLs contain three major elements: a cullin scaffold, a RING finger protein (RBX1 or RBX2) that recruits a ubiquitin‐charged E2 enzyme, and a substrate adaptor that places substrates in proximity to the E2 enzyme to facilitate ubiquitin transfer.^[^ ^10^ ^]^ Seven cullin (1, 2, 3, 4A, 4B, 5 and 7)‐RING complexes interact with distinct sets of adaptor modules, forming about 200 unique CRL complexes in total. Importantly, Cullin is modified by Nedd8 and is critical for its activity.^[^ ^11^ ^]^ We previously identified that CSN6 enhanced neddylation of culllin‐1 and facilitated autoubiquitination/degradation of Fbxw7α, a component of CRL involved in Myc ubiquitination, thereby stabilizing Myc.^[^ ^9e^ ^]^ Besides, we also found that CSN6 promotes tumorigenesis in mice through directly stabilization of MDM2.^[^ ^12^ ^]^ However, targets of CSN6 lack systematic proteomics assay and the mechanism regulation warrants further investigation.

Here we find that CSN6 is highly expressed in HCC and correlates with poor survival. CSN6 promotes HCC cancer cell growth in vitro and in vivo through YAP1 nuclear translocalization and activation. With IP‐mass spectrometry and quantitative proteomics assay, we identified 68 potential CSN6 direct targets and found that CSN6 directly regulates HMGCS1 protein stability. Further study shows that CSN6 stabilizes HMGCS1 protein by preventing SPOP‐mediated ubiquitination and degradation of HMGCS1. Mechanistic study shows that MDM2 is a new E3 ligase of SPOP. Our study demonstrates that CSN6‐HMGCS1‐YAP1 pathway is a predictive biomarker in HCC and can be targeted for NAFLD related HCC treatment.

## 2 Results

### 2.1 CSN6 Is Highly Expressed in HCC, and Csn6 Liver‐Specific Knockout Attenuated Liver Tumor Growth

**Figure 1:**
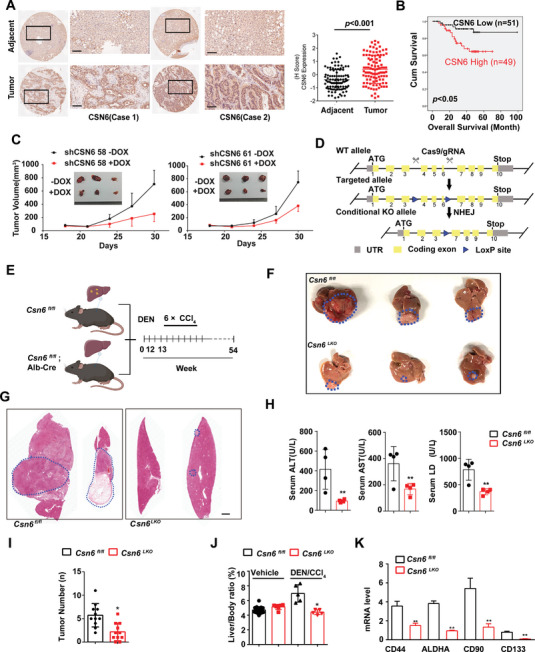
CSN6 promotes HCC growth and correlates with poor survival. A) Representative image of CSN6 IHC staining in human liver cancer and adjacent normal liver tissue. Scale bars, 100 µm. Quantitative CSN6 expression was shown in paired HCC tissue and adjacent normal tissue (right panel). Data are presented as mean ± SD. B) Kaplan‐Meier survival curves of overall survival duration based on CSN6 expression in human HCC tissue microarray. C) Impact of DOX‐induced shCSN6 on tumor growth of Huh‐7 xenograft tumors. Tumor volume was measured. The data are presented as the means ± s.d. *n* = 5; **, *p* < 0.01. D) Schematic depiction of generating *Csn6* conditional knockout (KO) mouse model. E) Time line of *Alb*‐ *Cre* mediated liver‐specific *Csn6* knockout (KO) mouse treated with DEN/CCl_4_ treatment. *Csn6* ^fl/fl^ mice (*n* = 10) and *Csn6* ^LKO^mice (CSN6^LKO^, *n* = 10) were injected with DEN (100 mg kg^−1^, i.p.) at the age of 12 weeks followed by six injections of CCl_4_ (0.5 mL kg^−1^, i.p.) and sacrificed 12.5 months after DEN. Scale bar, 2 mm. F) Gross morphology of DEN/CCl_4_‐challenged *Csn6* ^fl/fl^ and *Csn6* ^LKO^ mice. G) H&E staining of DEN/CCl_4_‐challenged *Csn6* ^fl/fl^ and *Csn6* ^LKO^ mice. H) Serum level of ALT, AST, and LDH from indicated mice was measured after DEN/CCl_4_ treatment. **, *p* < 0.01. I) Tumor number from indicated mice were determined. *, *p* < 0.05; **, *p* <0 .01. J) Liver body ratios from indicated mice were determined. *, *p* < 0.05. K) Liver cancer marker genes were determined by qPCR. *n* = 3; **, *p* < 0.01.

We first analyzed CSN6 expression with quantitative PCR from 25 paired samples of liver cancer tissue and adjacent normal tissue and found that CSN6 expression in cancer tissue was significantly higher than that in normal tissue (Figure S1A,B, Supporting Information). With bioinformatics analysis, we found that CSN6 is upregulated in HCC and correlates with poor survival from GSE14520 and TCGA data set (Figure S1C, Supporting Information). Our cohort studies again confirm that CSN6 is upregulated in tumor tissue when compared with adjacent normal tissues (**Figure** **1**A). Kaplan‐Meier analysis from our cohort showed that high CSN6 correlates with poor survival (Figure 1B). With cell confluence assay and colony formation assay, we find that knockdown (KD) of CSN6 significantly inhibited tumor growth in HCC cell line in vitro (Figure S1D–F, Supporting Information). With subcutaneous xenograft mouse model, CSN6 KD hinders tumor growth, accompanied with decreased Ki‐67 expression (Figure 1C and Figure S1G, Supporting Information). Thus, we generated *Csn6* ^fl/fl^ mice with CRISPR/Cas9 by targeting exons 4–6 and verified the genotype with qPCR analysis (Figure 1D and Figure S1H, Supporting Information). Combination of diethyl nitrosamine (DEN) and the hepatotoxin carbon tetrachloride (CCl_4_) treatment initiates HCC in mice, which incorporates chronic injury, inflammation, fibrogenesis, and demonstrates several features of human HCC (Figure 1E). Importantly, *Csn6* liver‐specific knockout (*Csn6* ^fl/fl^; *Alb‐Cre* (*Csn6* ^LKO^)) mice attenuated tumor growth after DEN/CCl_4_ treatment (Figure 1F,G), with significantly decreased liver injury markers including alanine amino transferase (ALT), aspartate amino transferase (AST), and lactate dehydrogenase (LDH) when compared with *Csn6* ^fl/fl^ mice (Figure 1H). *Csn6* ^LKO^ mice also showed less tumors, smaller tumor size, smaller ratios of liver/body weight and decreased liver cancer markers expression, such as CD44 and ALDHA, when compared with *Csn6* ^fl/fl^ mice (Figure 1I–K). IHC staining in *Csn6* ^LKO^ mice also demonstrated that tumor proliferation markers Ki‐67 and ALDHA were all significantly decreased in *Csn6* ^LKO^ mice ( Figure S1I, Supporting Information). Together, these results suggested that high CSN6 expression is important for HCC growth and correlates with poor survival.

### 2.2 CSN6 Promotes YAP1 Activation in HCC

**Figure 2:**
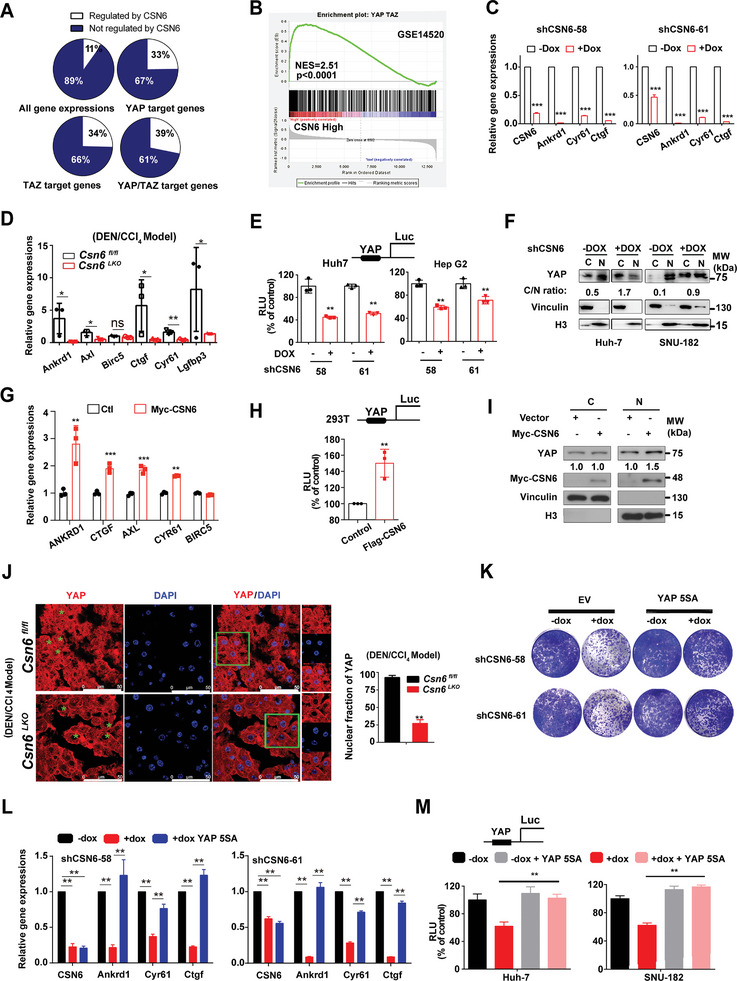
CSN6 promotes YAP1 transcriptional activity. A) 33%–39% of genes activated by YAP/TAZ are also regulated by CSN6. Huh7 cell line was infected with DOX‐inducible shCSN6 RNA. RNA‐seq was performed. B) Gene set enrichment analysis (GSEA) of GSE14520. GSEA plot of YAP signaling pathway signature correlated with CSN6 high related genes. NES, normalized enrichment score. C) CSN6 KD led to decreased gene expression of YAP targets. qPCR was performed for indicated genes in CSN6 KD Huh‐7 cell line. *n* = 3; ***, *p* < 0.001. D) Relative gene expression of YAP1 targets in liver tissues of *Csn6* ^fl/fl^ and *Csn6* ^LKO^mice. *, *p* < 0.05; **, *p* < 0.01; ns, not significant. E) Silencing CSN6 decreased YAP transcriptional activity with luciferase reporter gene assay (8XGTII‐lux). Luciferase activity assay of YAP in DOX‐induced KD of CSN6 in indicated cancer cell lines. Cells were transfected with vector or 8XGTII‐lux. RLU, Relative Luciferase Units. *, *p* < 0.05; **, *p* < 0.01; *n* = 3. F) Nuclear fraction assay of YAP1 shows silencing CSN6 increased YAP1 translocation from the nucleus to the cytoplasm. G) CSN6 overexpression led to increased gene expression of YAP targets in MHCC‐97H cell line. **, *p* < 0.01; ***, *p* < 0.001; *n* = 3. H) CSN6 overexpression enhanced YAP transcriptional activity with luciferase reporter gene assay (8XGTII‐lux). **, *p* < 0.01; *n* = 3. I) Nuclear fraction assay of YAP1 shows that CSN6 overexpression increased YAP1 translocation from the cytoplasm to the nucleus. J) Immunofluorescence staining of YAP1 in liver tissues from DEN/CCl_4_‐treated *Csn6* ^fl/fl^ and *Csn6* ^LKO^ mice. Percentage of YAP1 nuclear localization was quantitated. K–M) CSN6 KD attenuated colony formation, YAP1 target expression, and YAP1 transcriptional activities. YAP‐5SA overexpression reversed these effects caused by CSN6 KD.

To gain mechanistic insights into the tumor‐promoting effect of CSN6, we performed RNA‐seq experiments. CSN6 KD represses the expression of YAP/TAZ target genes (**Figure** **2**A). With GSEA analysis, we found that CSN6 positively correlates with YAP/TAZ target genes expression (Figure 2B). With quantitative PCR assay, we showed that CSN6 ablation attenuates YAP1 target gene expression in CSN6 KD cell line and in liver tissues of *Csn6* ^LKO^ mice (Figure 2C,D). YAP activation always needs to translocate to nucleus and function as transcription co‐factor with TEAD. With transcriptional activity assay, we found that CSN6 ablation repressed YAP1‐TEAD transcriptional activity (Figure 2E). Furthermore, CSN6 KD reduced YAP1 nuclear localization (Figure 2F and Figure S2A, Supporting Information). CSN6 overexpression increases YAP1 target gene expression, enhancing YAP1‐TEAD transcriptional activity and promoting YAP1 nuclear localization (Figure 2G–I). Congruently, we found that YAP1 nuclear translocation is compromised in liver cancer tissues of *Csn6* ^LKO^ mice (Figure 2J). To further confirm that CSN6‐mediated tumor growth is YAP1‐dependent, we overexpressed YAP1‐5SA, a constitutive‐active YAP1, and found that YAP1‐5SA can rescue CSN6 KD‐mediated YAP1 target gene suppression, colony formation inhibition, and transcriptional attenuation (Figure 2K–M). Taken together, our data demonstrate that CSN6 promotes YAP1 nuclear translocation and YAP1 transcriptional activity to facilitate tumor growth.

### 2.3 Proteomics Demonstrates HMGCS1 as a Target of CSN6 in HCC

**Figure 3:**
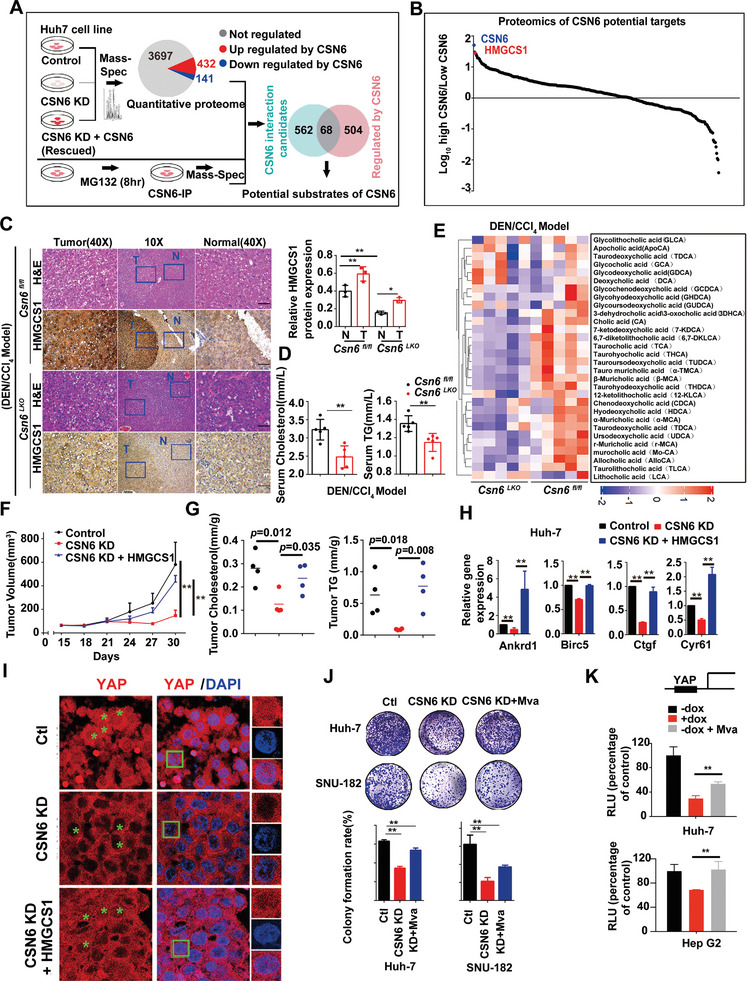
CSN6 promotes YAP1 activation through HMGCS1 mediated mevalonate metabolism. A) A two‐step screen strategy identified CSN6 substrate in liver cancer. Huh7 cells were transduced with DOX‐inducible shCSN6 or CSN6 rescued lentivirus. CSN6 protein was also immunoprecipitated in the presence of MG132 for 8 h. Mass‐spectrometry was performed to identify CSN6 regulated proteins and associated proteins. B) CSN6 direct substrates were identified in liver cancer by proteomics. The target proteins were ranked according to DEGs (differently expressed genes) regulated by CSN6. Each dot represents one candidate protein. C) H&E staining and immunohistochemistry analysis of HMGCS1 expression level in liver tissues of DEN/CCl_4_‐treated *Csn6* ^fl/fl^ and *Csn6* ^fl/fl^; *Alb‐Cre* (*Csn6* ^LKO^) mice. Area of tumor and adjacent normal tissue was noted. Scale bar, 50 µm. Quantification of HMGCS1 expression level was shown on the right panel. *, *p* < 0.05; **, *p* < 0.01; *n* = 3. D) Levels of cholesterol and triglycerides (TG) in serum of DEN/CCl_4_‐treated *Csn6* ^fl/fl^ and *Csn6* ^LKO^ mice were measured. **, *p* < 0.01; *n* = 3. E) Levels of various BA measured in livers of *Csn6* ^fl/fl^ and *Csn6* ^LKO^ mice after DEN/CCl_4_ treatment was presented as a heat map. F) Tumor growth curves of Huh‐7 (1 × 10^6^) liver cancer cells transfected with indicated constructs. Cells were subcutaneously injected into nude mice (*n* = 5). Tumors were collected at the end of the experiments. **, *p* < 0.01. G) Cholesterol and TG of tumor tissues were measured. H) YAP target genes of tumor tissues were measured. **, *p* < 0.01. I) Immunofluorescence staining of YAP1 in tumors were presented. HMGCS1 increased YAP1 nuclear translocation in the presence of CSN6 KD. Scale bar, 50 µm. J) CSN6 KD attenuated colony formation and mevalonate(Mva) reversed the effects caused by CSN6 KD. **, *p* < 0.01; *n* = 3. K) CSN6 KD attenuated YAP1 transcriptional activities. Mevalonate (Mva) reversed the effects caused by CSN6 KD. **, *p* < 0.01; *n* = 3.

To identify the potential targets for CSN6 in HCC, we performed quantitative proteomic analysis based on three groups: control group, shCSN6 group and CSN6 rescued group. We identified approximately 4200 proteins per sample. 432 proteins were upregulated, and 141 proteins were downregulated by CSN6 expression. At the same time, we performed CSN6 immunoprecipitation and identified 630 candidate proteins interacting with CSN6 (**Figure** **3**A and Figure S3A, Supporting Information). 68 proteins are potential direct target of CSN6 as they are within the intersection of the CSN6‐binding proteins and CSN6‐regulated proteins (Figure S3B, Supporting Information). We found that HMGCS1, a mevalonate metabolism enzyme, is at the top rank of CSN6‐regulated substrates (Figure 3B). HMGCS1 converts acetyl‐CoA to HMG‐CoA and promotes mevalonate metabolism.^[^ ^13^ ^]^ To confirm the proteomic results, we found that CSN6 KD in liver cancer led to reduced steady‐state expression of HMGCS1 (Figure S3C, Supporting Information). Congruently, *Csn6* ^LKO^ mice have attenuated HMGCS1 protein expression, but not HMGCR, in liver tissues when compared with *Csn6* ^fl/fl^ mice (Figure 3C and Figure S3D, Supporting Information). Also, *Csn6* ^LKO^ mice demonstrated lower cholesterol/TG levels in serum when compared with *Csn6* ^fl/fl^ mice (Figure 3D). Given that bile acids (BA) have been linked to cholesterol metabolism, NASH progression, and HCC development,^[^ ^14^ ^]^ the profile of hepatic BA was examined with mass spectrometry to characterize the possible link. Significantly, the total BA content in liver was decreased in *Csn6* ^LKO^ mice when compared with *Csn6* ^fl/fl^ mice (Figure 3E). Our animal experiments further showed that CSN6 and HMGCS1 are both upregulated in premalignant lesions (24 and 72 h) and HCC nodules (12 months) during malignant transformation with DEN/CCl_4_ (Figure S3E, Supporting Information). These results demonstrated that deregulation of HMGCS1‐mediated cholesterol metabolism contributes to HCC development.

Rho family of GTPase modulates actin cytoskeleton and positively controls YAP/TAZ activity.^[^ ^15^ ^]^ Previous study suggested that mevalonate pathway also controls YAP and TAZ activation through Rho GTPase, which is independent of LATS1/2 kinases.^[^ ^16^ ^]^ To further clarify the relationship between CSN6 regulated YAP activation and mevalonate metabolism, we then demonstrated that HMGCS1 overexpression can revert CSN6 KD‐mediated tumor growth inhibition in subcutaneous xenograft model (Figure 3F). Further, we collected tumor tissues and found that HMGCS1 overexpression rescued CSN6 KD‐mediated decreased cholesterol and TG levels, and YAP1 target gene expression (Figure 3G,H). Moreover, CSN6 KD‐mediated YAP1 cytoplasmic translocation is also reverted by HMGCS1 expression (Figure 3I). In addition, mevalonate metabolite can rescue colony formation reduction and YAP1 transcriptional activity repression caused by CSN6 KD (Figure 3J,K). Taken together, these data showed that CSN6‐HMGCS1 axis regulates mevalonate metabolism, promoting YAP1 nuclear translocation and transcriptional activity to facilitate tumor progression.

### 2.4 CSN6 Promotes HMGCS1 Stabilization by Attenuating HMGCS1 Ubiquitination

**Figure 4:**
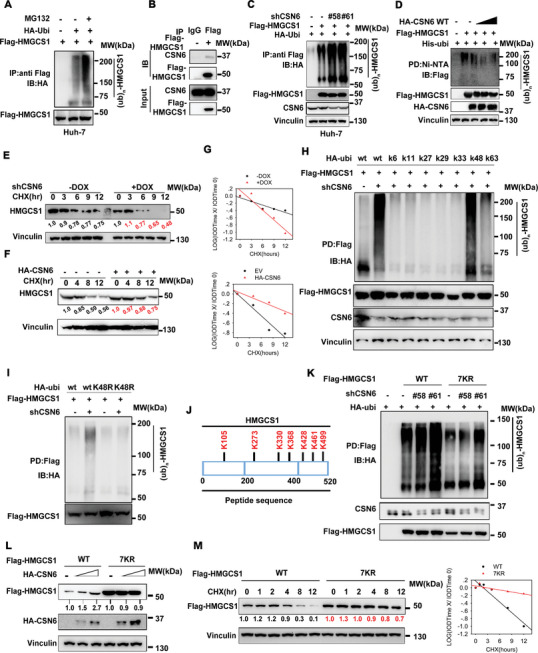
CSN6 attenuates K48‐mediated HMGCS1 ubiquitination. A) Poly‐ubiquitination assay of HMGCS1. Immunoblot analysis of poly‐ubiquitinated HMGCS1 in 293T cells transfected with the indicated constructs and treated with MG132 for 6 h. B) Immunoblot analysis of the indicated proteins from immunoprecipitates (IP) obtained from 293T cells with MG132 treatment for 6 h. CSN6 associated with HMGCS1. **C**) CSN6 KD increased HMGCS1 poly‐ubiquitination in 293T cells. Immunoblot analysis of poly‐ubiquitinated HMGCS1 in 293T cells transfected with the indicated constructs and treated with MG132 for 6 h. D) CSN6 enhanced HMGCS1 poly‐ubiquitination in a dose‐dependent manner. Cells transfected with indicated constructs were treated with MG132 (10 × 10^‐6^ m) 6 h before harvest. The cell lysates were pulled down (PD) with nickel beads (Ni‐NTA) and immunoblotted with indicated antibodies. E,F) CSN6 overexpression decreased HMGCS1 protein turnover rates. CSN6 KD accelerated HMGCS1 protein turnover rate. Representative immunoblots showing HMGCS1 protein turnover rate in cells treated with cycloheximide (CHX, 60 µg mL^‐1^), in the presence or absence of CSN6 (Top). G) Quantification data were shown. IOD, integrated optical density. The relative density of CSN6 was normalized to Vinculin and then normalized to the *t* = 0 control. H,I) CSN6 KD enhanced HMGCS1 K48‐linked ubiquitination. Cells transfected with indicated constructs were treated with MG132 (10 × 10^‐6^ m) 6 h before harvest. The cell lysates were pulled down (PD) with M2 beads and immunoblotted with indicated antibodies. J) The predicted ubiquitination sites of HMGCS1 protein. K) HMGCS1‐7KR mutant is resistant to ubiquitination. Cells transfected with indicated constructs were treated with MG132 (10 × 10^‐6^ m) 6 h before harvest. The cell lysates were pulled down (PD) with M2 beads and immunoblotted with indicated antibodies. L) The HMGCS1‐7KR mutant is more stable than HMGCS1‐WT in the presence of CSN6 based on steady‐state expression studies. **M** Representative immunoblots showing HMGCS1 7KR mutant protein turnover rate in 293T cells. The HMGCS1 7KR mutant has a slower turnover rate.

Given that CSN6 regulated targeted protein through ubiquitin‐mediated degradation, we ask if CSN6 regulates HMGCS1 ubiquitination. We found that MG132 increases HMGCS1 protein expression and that HMGCS1 is ubiquitinated (**Figure** **4**A and Figure S4A, Supporting Information). Co‐IP showed the association between CSN6 and HMGCS1 (Figure 4B). Importantly, CSN6 KD efficiently increased the polyubiquitination level of HMGCS1 (Figure 4C), while CSN6 overexpression reduced HMGCS1 polyubiquitination (Figure 4D). Overexpression of CSN6 leads to a slower turnover rate of HMGCS1, while CSN6 KD accelerated the turnover rate of HMGCS1 (Figure 4E–G). In addition, HMGCS1 is highly tagged with wild‐type or K48 ubiquitin (ubiquitin mutant that contains only K48 lysine) under CSN6 KD, indicating that CSN6 is involved in decreasing K48‐link ubiquitination of HMGCS1 (Figure 4H). Further, HMGCS1 is resistant to K48R‐mediated ubiquitination under CSN6 KD, we then confirmed that CSN6 regulated HMGCS1 ubiquitination is through K48 link (Figure 4I). Several potential ubiquitination sites were predicted (lysine residues) in HMGCS1 by UbPred (Figure 4J). Single lysine to arginine mutants of HMGCS1 failed to reduce their ubiquitination level (Figure S4B, Supporting Information). However, HMGCS1 ubiquitination was attenuated if all seven lysine residues were simultaneously mutated to arginine (7KR mutant) under CSN6 KD (Figure 4K). Therefore, HMGCS1 (7KR) is more stable than HMGCS1‐WT even when CSN6 is not overexpressed (Figure 4L). Consistently, HMGCS1 (7KR) has decelerated turnover rate when compared with HMGCS1‐WT (Figure 4M). Together, CSN6 reduced K48‐linked ubiquitination on multiple sites of HMGCS1, thereby stabilizing HMGCS1 and promoting HCC outgrowth.

### 2.5 E3 Ligase SPOP Is Involved in Regulating HMGCS1 Ubiquitination

**Figure 5:**
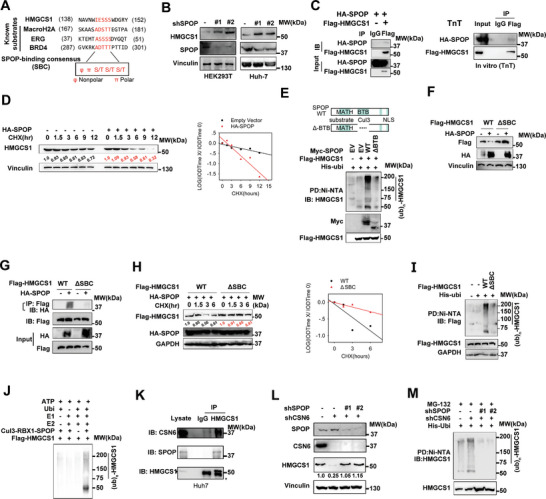
SPOP is involved in HMGCS1 dysregulation. A) Amino acid sequence alignment of putative SPOP binding consensus (SBC) motifs in HMGCS1. MacroH2A, ERG and BRD4 are known SPOP substrates containing well‐characterized SBC motifs. B) SPOP KD increased HMGCS1 steady‐state expression in 293T and Huh‐7 cell line. C) Immunoblot analysis of immunoprecipitates obtained from 293T cells transfected with indicated constructs and treated with MG132 for 6 h (left panel). HMGCS1 interacts with SPOP in vitro (right panel). Indicated constructs were transcribed and translated using TnT kit. Protein‐protein interaction was assayed by co‐IP experiments using indicated antibodies. HMGCS1 interacts with SPOP. D) Representative immunoblots showing HMGCS1 protein turnover rate in 293T cells treated with CHX, in the presence of SPOP. SPOP overexpression increased the turnover rate of HMGCS1. E) Immunoblot analysis of HMGCS1 ubiquitination from 293T cells transfected with the indicated constructs and treated with MG132 for 6 h. F) Immunoblots showing HMGCS1 steady‐state expression in indicated 293T cells transfected with HMGCS1‐ΔSBC. HMGCS1‐ΔSBC is resistant to SPOP‐mediated degradation. G) HMGCS1‐WT, but not HMGCS1‐ΔSBC, interacts with SPOP based on IP assay. H) SPOP overexpression cannot increase HMGCS1‐ΔSBC protein turnover rates in 293T cells with CHX treatment. I) HMGCS1‐ΔSBC is resistant to poly‐ubiquitination assayed by ubiquitination assay. J) Immunoblot of HMGCS1 poly‐ubiquitination in an in vitro ubiquitination assay by the CUL3‐RBX1‐SPOP E3 ligase complex. K) HMGCS1 interacts with SPOP and CSN6 in liver cancer cell. Immunoblot analysis of the indicated proteins from immunoprecipitates obtained from Huh‐7 cells treated with MG132 for 6 h. L) CSN6 KD led to reduced HMGCS1 steady‐state expression via upregulating SPOP. Immunoblot analysis of indicated proteins in 293T cells transfected with the indicated constructs. M) CSN6 KD‐mediated increased ubiquitination of HMGCS1 is SPOP‐dependent. Immunoblot analysis of poly‐ubiquitinated HMGCS1 in 293T cells transfected with the indicated constructs and treated with MG132 for 6 h.

We next sought to identify the E3 ubiquitin ligase that targets HMGCS1. The primary amino acid sequence of HMGCS1 protein contains a conserved consensus sequence that is a SPOP‐binding consensus (SBC) motif^[^ ^17^ ^]^ located at (133‐157aa) region (**Figure** **5**A and Figure S4C, Supporting Information). We found that SPOP overexpression attenuated HMGCS1 steady‐state expression (Figure S4D, Supporting Information). SPOP KD increased the steady‐state expression of HMGCS1 (Figure 5B). We performed in vivo (cell lysate) and in vitro (protein produced from TnT) co‐IP to demonstrate that HMGCS1 interacted with SPOP (Figure 5C). Also SPOP overexpression increases the turnover rate of HMGCS1 (Figure 5D). SPOP is a Cul3‐based E3 ligase and binds with Cul3 via its BTB domain. We found that SPOP‐ΔBTB mutant cannot increase HMGCS1 polyubiquitination when compared with SPOP‐WT (Figure 5E). We found that Cul3 is indeed involved in HMGCS1 steady‐state expression and ubiquitination (Figure S4E,F, Supporting Information). HMGCS1 protein with deletion of this putative SBC motif (HMGCS1‐ΔSBC) not only abolished SPOP binding, SPOP‐mediated ubiquitination and degradation, but also substantially reduced its turnover rate (Figure 5F–I). We further found that SPOP‐Cul3‐RBX1 complex catalyzed the ubiquitination of HMGCS1 in vitro (Figure 5J). These results prompted us to investigate how CSN6 is involved in SPOP‐mediated HMGCS1 ubiquitination. Notably, IP showed the association among CSN6, SPOP and HMGCS1 (Figure 5K). Importantly, we found that CSN6 KD mediated elevation of SPOP, which in turn translates into reduced steady‐state expression of HMGCS1 and enhanced ubiquitination of HMGCS1 (Figure 5L,M). Thus, CSN6 antagonizes SPOP‐mediated HMGCS1 polyubiquitination.

### 2.6 CSN6‐MDM2 Axis‐Mediated SPOP Ubiquitination

**Figure 6:**
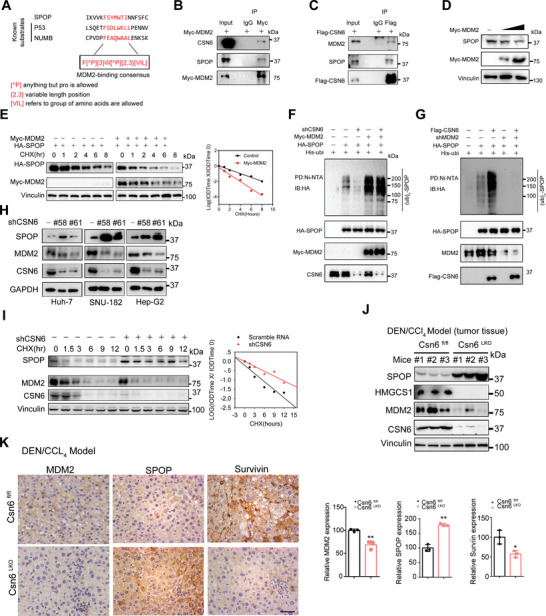
CSN6‐MDM2‐SPOP axis is involved in HMGCS1 dysregulation. A) Amino acid sequence alignment of putative MDM2 binding consensus motifs in SPOP. P53 and NUMB are known MDM2 substrates containing well‐characterized consensus motifs. B) Protein–protein interaction was assayed by co‐IP experiments using indicated antibodies. MDM2 interacts with CSN6 and SPOP. **C)** Protein‐protein interaction was assayed by co‐IP experiments using indicated antibodies. CSN6 interacts with MDM2 and SPOP. D) MDM2 overexpression decreases SPOP expression. E) Representative immunoblots showing SPOP protein turnover rate in 293T cells treated with CHX, in the presence of MDM2. MDM2 overexpression increased the turnover rate of SPOP. F,G) Immunoblot analysis of SPOP ubiquitination from 293T cells transfected with the indicated constructs and treated with MG132 for 6 h. H) Representative immunoblots showing SPOP and MDM2 expression in cancer cells transfected with shCSN6. Silencing CSN6 increased SPOP steady‐state expression in liver cancer cell lines. I) Representative immunoblots showing SPOP and MDM2 protein turnover rate in 293T cells treated with CHX (left), in the presence of shCSN6. Silencing CSN6 decreased SPOP protein turnover rates. J) Immunoblot analysis of indicated proteins in liver tissues of *Csn6* ^fl/fl^ and *Csn6* ^LKO^ mice. K) CSN6 depletion leads to decreased MDM2 expression and increased SPOP protein expression. Scale bar, 50 µm. *, *p* < 0.05; **, *p* < 0.01; *n* = 3.

To figure out the mechanistic intersection between CSN6 and SPOP, we find that SPOP contains a conserved MDM2 binding motif (**Figure** **6**A). We previously found that CSN6 stabilizes MDM2 via blockade of its auto‐ubiquitination.^[^ ^12^ ^]^ Thus we reasoned that MDM2 might function as an E3 ligase of SPOP. Immunoprecipitation assay shows that CSN6 interacts with MDM2 and SPOP (Figure 6B,C). MDM2 overexpression decreased the expression of SPOP and increased the turnover rate of SPOP (Figure 6D,E). With ubiquitination assay, CSN6 KD decreased ubiquitination of SPOP (Figure 6F). However, MDM2 overexpression enhanced the ubiquitination of SPOP (Figure 6F). On the other hand, CSN6 overexpression‐mediated SPOP ubiquitination is dependent on MDM2 expression (Figure 6G). All these data suggested that CSN6 promotes SPOP degradation via E3 ligase MDM2. Moreover, we found that CSN6 KD increased the steady‐state expression of SPOP protein and reduced the turnover rate of SPOP (Figure 6H,I). Concurrent decreased MDM2 expression and increased MDM2 turnover rate were observed, suggesting the interplay between CSN6, MDM2 and SPOP. Under physiological condition, *Csn6* ^LKO^ liver cancer tissue demonstrated higher SPOP expression, less expression of MDM2, HMGCS1 and survivin (YAP1 target gene) when compared with *Csn6* ^fl/fl^ liver cancer tissue (Figure 6J,K), suggesting that CSN6‐MDM2 axis‐mediated SPOP ubiquitination and consequential HMGCS1 stabilization can be recapitulated in *Csn6* ^LKO^ mouse liver cancer.

### 2.7 Targeting CSN6‐HMGCS1 Axis to Suppress Tumor Growth in NAFLD Related HCC

**Figure 7:**
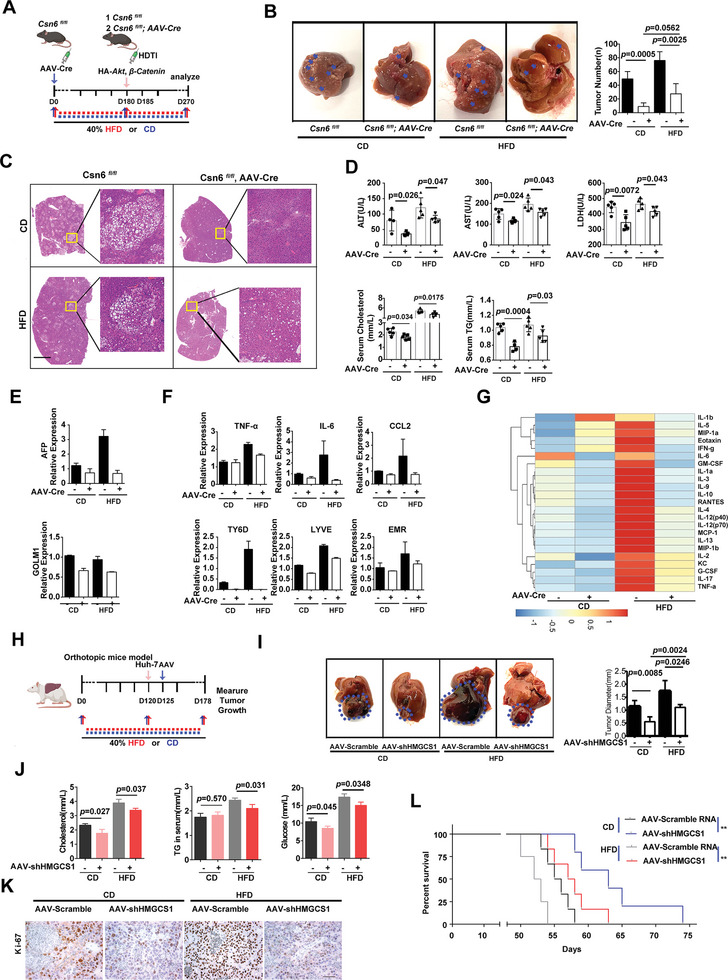
Activated CSN6‐HMGCS1 signaling provides vulnerability for HFD‐induced liver tumor growth. A) Schematic depiction of generating liver‐specific *Csn6* ^LKO^ (HDTI) mice employing hydrodynamic tail vein injection (HDTI) strategy. Mice were grouped into CD and HFD based on diet feeding. CD, control diet; HFD, high fat diet. AAV‐Cre; AAV8‐Cre recombinase. B) Representative macroscopic photographs of livers with tumor growth in indicated groups. Arrow heads indicate tumor nodules of HCC. The average liver tumor numbers in each group were shown. C) Representative H&E staining of liver tissues in indicated groups. Scale bar, 2 mm. D) Quantification of serum cholesterol, TG, ALT, AST and LDH in each group of mice. E) qPCR quantification of mRNA of indicated HCC markers in tumor tissues. *, *p* < 0.05; **, *p* < 0.01; *n* = 3. F qPCR quantification of mRNA of indicated inflammatory target genes in tumor tissues. *, *p* < 0.05; **, *p* < 0.01; ns, not significant; *n* = 3. G) Cytokine array screening of the serum from indicated group of mice by luminex technology (Plex pro mouse Cytokine). The cytokine levels were presented as a heat map. H) Schematic depiction of generating orthotopic liver cancer model using Huh‐7 cells infected with AAV‐shHMGCS1 under CD or HFD conditions. I) Representative macroscopic photographs of liver with tumor growth in indicated group from orthotopic Huh‐7 liver cancer model. The average liver tumor diameter was shown. J) Quantification of serum cholesterol, TG, and glucose in indicated groups of mice from orthotopic Huh‐7 liver cancer model. K) Representative Ki‐67 staining from AAV‐shHMGCS1 treated Huh‐7 orthotopic cancer model. Scale bar, 50 µm. L) Survival curve generated from indicated orthotopic Huh‐7 liver cancer model. **, *p* < 0.01.

Mevalonate metabolism is used in multiple anabolic processes that support cancer cell growth and proliferation especially when cholesterols are abundant.^[^ ^18^ ^]^ High fat diets (HFD) lead to more cholesterol synthesis and can promote tumor growth in mice models via activating YAP/TAZ pathway.^[^ ^19^ ^]^ We previously found that AKT can directly phosphorylate CSN6 at S60.^[^ ^9a^ ^]^ Thus, we employed HA‐AKT, HA‐β‐Catenin, and SB transposon to deliver these constructs into liver of *Csn6* ^fl/fl^ C57 mice by hydrodynamic tail vein injection (HDTI) to establish CSN6 high liver cancer model. *Csn6^LKO^* (HDTI) was established by extra treatment with AAV‐*cre* to knockout *Csn6* in these mouse liver cancer model (**Figure** **7**A). *Csn6* ^fl/fl^ (HDTI) and *Csn6* ^LKO^ (HDTI) mice were grouped and fed with HFD or control diet (CD) for experimental design (Figure 7a). Obviously, HFD leads to faster liver tumor growth when compared with CD (Figure 7B). Importantly, *Csn6* ^LKO^ mice have attenuated liver tumor growth in HFD or CD mice (Figure 7B,C), further suggesting that CSN6 is critical for liver tumor growth and that CSN6 loss leads to limited tumor growth even in cholesterol abundant environment (under HFD condition). Also, *Csn6* ^LKO^ (HDTI) mice have decreased level of ALT, AST, LDH, cholesterol and TG in both CD and HFD conditions (Figure 7D). Consistently, *Csn6* ^LKO^ (HDTI) mice have compromised HCC marker expression (AFP, GOLM1) (Figure 7E). RT‐PCR and cytokines array showed that *Csn6* ^LKO^ (HDTI) mice have decreased inflammatory activation elevated by HFD in liver microenvironment (Figure 7F,G). Moreover, in orthotopic liver cancer model established by injecting Huh7 cells infected with AAV‐shHMGCS1 into liver, HMGCS1 KD diminished liver tumor growth and proliferation under both CD and HFD conditions (Figure 7H,I). Also, HMGCS1 KD mice have decreased level of cholesterol, TG, glucose and Ki‐67 in both CD and HFD conditions (Figure 7J,K). Significantly, HMGCS1 KD improved tumor survival in this orthotopic liver cancer model under both CD and HFD conditions (Figure 7L). These results suggested that CSN6/HMGCS1 axis provides targetable vulnerability in liver cancer accompanied with non‐alcoholic steatohepatitis and inflammation.

### 2.8 CSN6/HMGCS1 Overexpression Provides Targetable Vulnerability in HCC

**Figure 8:**
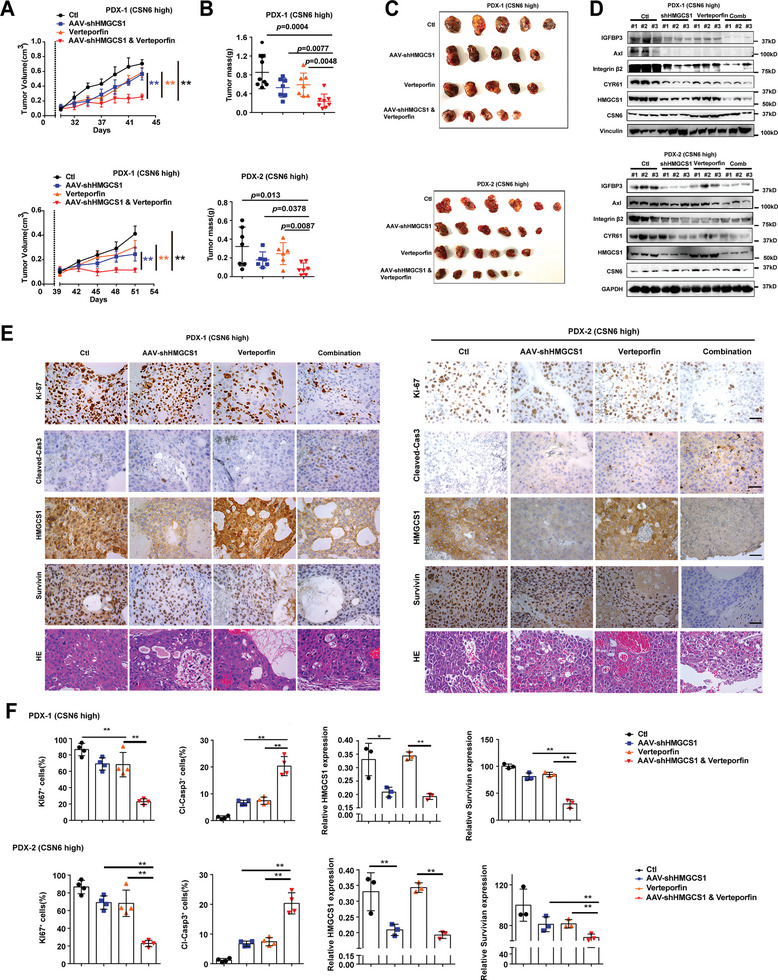
Targeting HMGCS1‐YAP1 axis suppresses HCC growth. A) Combination targeting YAP1 and HMGCS1 inhibits tumor growth in PDX (patient derived xenograft) models. Human liver cancer tissues were grown as tumor xenografts in NCG mice. Growth curves demonstrate the proliferation of PDX tumors in each indicated treatment group: AAV‐shHMGCS1(1 × 10^12^ v g mL^‐1^, 5 µL), verteporfin (100 mg kg^−1^ per day, I.P.), or a combination of both. Data are presented as mean ± SD. **, *p* < 0.01. B) Tumor mass was measured for each group. C) Representative Images of the PDX tumors were harvested at the end of the experiment. D) Immunoblot analysis of protein levels of IGFBP3, Axl, Integrin β2, CYR61, HMGCS1 and CSN6 in the PDX tumor tissues. E) Representative IHC images of Ki‐67, cleaved‐Caspase3, HMGCS1, survivin and HE staining in PDX tumors from the indicated treatment groups were shown. Scale bars, 50 µm. F) Quantification of the indicated protein expressions was shown. **, *p* < 0.01; *n* = 3.

To further explore the translational significance of these findings, we established HCC PDX (patient derived xenograft) models from tumor tissues of HCC patients with high CSN6/HMGCS1 expression. PDX tumors that received verteporfin (YAP inhibitor) or AAV‐shHMGCS1 monotherapy showed a modest reduction in terms of tumor volume and tumor weight. Importantly, the combination of verteporfin and AAV‐shHMGCS1 treatment was more efficient in hindering tumor growth than verteporfin or AAV‐shHMGCS1 alone in CSN6/HMGCS1 high PDX as demonstrated by reduced tumor volume (**Figure** **8**A) and reduced tumor weight (Figure 8B,C). With western blot assay, combination treatment diminished expression of YAP1 transcriptional targets, including IGFBP3, Axl, Integrin β2 and CYR61 (Figure 8D). IHC staining showed that combination treatment decreased survivin (YAP target), Ki‐67 staining, increased cleaved caspase‐3 staining, and attenuated HMGCS1 expression (Figure 8E,F). Together, YAP inhibitor plus shHMGCS1 as a combination therapy may be considered for therapeutic design in high CSN6/HMGCS1 HCC patients.

## 3 Discussion

MVA pathway dysregulation in liver cancer remain not well‐characterized. Here we systematically unearthed the MVA deregulation through CSN6‐HMGCS1‐YAP1 axis during HCC development (Figure S5, Supporting Information). Using various orthotopic mice cancer models, we uncovered that MVA pathway regulator HMGCS1 was essential to promote tumor progression and YAP activation in HCC. Mechanistically, we show that CSN6, and SPOP have activities in regulating HMGCS1 expression. CSN6 negatively regulates SPOP to stabilize HMGCS1, which activates YAP via mevalonate metabolism. Our results reveal that targeting CSN6‐HMGCS1‐YAP1 axis provides targetable vulnerability in NAFLD related cancer.

### 3.1 Roles of CSN6 in YAP Activation/Cholesterol Metabolism Are Involved in Liver Cancer

CSN6 is overexpressed and involved in drug resistance in CRC.^[^ ^20^ ^]^ However, its biological functions in HCC have not been unveiled. We demonstrate that CSN6 is elevated in HCC and correlates with poor progonosis. Alb‐specific knockout of *Csn6* studies demonstrates the critical role of *Csn6* in promoting the growth of liver cancer. Oncogenic activity studies indicate CSN6's impacts on mevalonate pathway and YAP activation. Our data fill a knowledge gap by identifying for the first time that CSN6 overexpression is critical during tumorigenesis in HCC. Significantly, we found that HMGCS1 is a target stabilized by CSN6. *Csn6* knock out manifests concurrent reduced expression of HMGCS1, leading to reduction of total cholesterol/bile acid. With impact on HMGCS1, CSN6 affects nuclear localization of YAP1. It was shown that mevalonate metabolism intermediate, geranylgeranyl pyrophosphate (GGPP), activates RhoA, thereby directly activating YAP1, the major nuclear mechanotransducer,^[^ ^20^ ^]^ to translocate into the nucleus and promote YAP/TAZ transcriptional cooperation with TEAD factors in the nucleus. Our CSN6 studies reveal that CSN6's impact on YAP1 activation results from its activity in stabilizing HMGCS1 and regulating mevalonate pathway.

### 3.2 CSN6‐MDM2 Signaling in Mitigating HMGCS1 Ubiquitination

Our data demonstrates for the first time that CSN6 regulates the stabilization of MDM2, which destabilizes E3 ligase SPOP, thereby antagonizing SPOP‐mediated HMGCS1 ubiquitination and degradation. Recurrent missense mutations in SPOP have been found in 5–10% of prostate and endometrial cancers in comprehensive genome sequencing studies. However, SPOP dysregulation in HCC has not been fully investigated. Our results show that lower SPOP protein expression also has prominent impact in HCC progression, which implies that SPOP deregulation at protein level (posttranscriptional regulation) may serve as a molecular marker in HCC.

Notably, MDM2 is characterized as an E3 ubiquitin ligase that binds and destabilizes SPOP. This result suggests that MDM2 has impacts in SPOP/HMGCS1 deregulated HCC. It has been shown that MDM2 oncoprotein ovexpression is frequently observed in hepatocellular carcinoma (HCC),^[^ ^21^ ^]^ Thus, MDM2's negative regulation on SPOP adds another layer of impact of MDM2 overexpression in HCC.

### 3.3 Efficacy of Targeting HMGCS1 in HFD‐Liver Cancer

Increased cholesterol biosynthesis and uptake is a hallmark of many cancers.^[^ ^22^ ^]^ As fast proliferating cells, cancer cells require high levels of cholesterol for cancer cell proliferation, migration, and invasion. However, dysregulation of mevalonate pathway enzymes in cancer has not been fully studied. Our animal cancer models have demonstrated promising effect in controlling HFD‐induced liver cancer growth by CSN6 deletion or knocking down HMGCS1. Animal experiments demonstrate important proof‐of concept in controlling CSN6 or HMGCS1 for HCC treatment such as NAFLD related cancer. Indeed, targeting abnormal cholesterol metabolism has been an appealing therapeutic strategy. As a mevalonate pathway regulator, HMGCS1 serves as an important target. Therefore, potential HMGCS1 inhibitors can be explored for the treatment of cancers including CSN6‐overexpressing HCC, such as Gypenosides^[^ ^23^ ^]^ and ligustilide.^[^ ^24^ ^]^ It will be an exciting avenue for using these inhibitors in cancer prevention or therapy; for examples, in HCC patients when CSN6/HMGCS1 expression was prognostically detrimental.

### 3.4 Combination of YAP Signaling Inhibitor Verteporfin and aav‐shHMGCS1 Treatment in Treating CSN6‐High HCC

Given that CSN6 stabilize HMGCS1 to regulate mevalonate pathway (cholesterol biosynthesis and biosynthesis of key isoprenoids) and can positively impact on YAP activation (important for pro‐cancer signaling pathway), it lends credence to the possibility that hindering YAP activation by verteporfin treatment plus attenuating HMGCS1 activity (aav‐shHMGCS1) might lead to a better synergistic effect in treating CSN6‐high HCC. Indeed, verteporfin (inhibits YAP1/TEAD interaction) plus aav‐shHMGCS1 as a combination treatment strategy for CSN6‐high HCC PDX demonstrated a better treatment efficacy than verteporfin or aav‐shHMGCS1 alone. These results bear important prognostic and therapeutic implications for improving therapeutic efficacy of HCC. Especially, (YAP/TAZ) activity in HCC cells is known to impair the verteporfin penetration into the cancer.^[^ ^25^ ^]^ HMGCS1 knockdown can at the same time reduce YAP activity to potentiate the penetration of verteporfin.

In summary, our studies demonstrate a link of CSN6 overexpression, MDM2‐mediated SPOP ubiquitination, HMGCS1 stabilization, mevalonate synthesis, YAP activation and HCC tumorigenicity. The impact of CSN6 in positively regulating HMGCS1 stability via hindering SPOP illustrates a new layer of regulation for the activation of YAP1 during tumorigenicity. Further developing drug compounds that interfere CSN6‐mediated HMGCS1 stabilization or inhibit HMGCS1 activity by small molecules can be further developed as a rational cancer therapy for CSN6‐overexpressing HCC.

## 4. Experimental Section

### Patients and Tissue Samples

Paraffin‐embedded samples of primary HCC (prepared as TMA), fresh frozen pared samples of primary HCC, and adjacent normal liver tissue were collected from the Cancer center of Sun Yat‐sen University and the Sixth affiliated hospital of Sun Yat‐sen University. The original immunohistochemistry slides were scanned by Aperio Versa (Leica Biosystems), which captured digital images of the slides. The Genie calculates an H‐score for regions selected by the pathologist. The receiver operating characteristic curve (ROC) was used to define the cut‐off point. All samples were collected with the patients’ written informed consent and approval from study center's institutional review board.

### Reagents and Plasmids

All transient transfections of plasmids and shRNA into cell lines followed the standard protocol for Lipofectamine2000 Transfection Reagent (Thermo Fisher, #11668019)

Treatment with inhibitors: MG132 (Selleck, S2619), MLN4924 (Selleck, S7109), cycloheximide (MD Bio, C012), Verteporfin (Selleck, S1786), mevalonic acid (Sigma, 79849). Doxycycline (Sigma, D9891).

### Antibodies

CSN6(Enzo, BML‐PW8295), HMGCS1(Proteintech, 17643‐1‐AP), HMGCR(SantaCruz, sc‐271595), Ki‐67(8D5)(Cell Signaling, #9449), cleaved‐Caspase 3 (Cell signaling, #9664s), ALDHA (Cell signaling, #54135s), GAPDH (Proteintech, 60004‐1), Vinculin (Cell signaling, #4650s), HA‐Tag(C29F4)(Cell Signaling,#3724S), Flag‐Tag (Sigma, F1804), Ni‐NTA agarose (Thermo, R90115), M2 beads(Sigma, A2220), Protein A/G agarose beads (50 µL,SantaCruz,SC‐2001), YAP1 (SantaCruz, sc‐101199; abcam, ab52771), SPOP (Proteintech, 16750‐1‐AP), YAP/TAZ targets antibody sampler kit (Cell Signaling, #56674).

### Cell Lines and Treatments

Huh‐7, Hep‐3B, Hep‐G2, MHCC‐97H, 293T and Hepa1‐6 were cultured in Dulbecco's modified Eagle's medium media supplemented with 10% FBS (fetal bovine serum). SNU‐182 cell was cultured in RPMI 1640 supplemented with 10% FBS. Cells were authenticated by STR profiling and are free from mycoplasma contamination. The confluence of the wells was determined using the Incucyte live cell analysis system (Sartorius).

### Lentivirus and Adeno‐Associated Virus (AAV)

To acquire lentiviral particles, 293T cells were cotransfected with 10 µg PLKO.1 shRNA construct, 5 µg of psPAX2 and 5 µg Pmd2.G. The supernatant containing viral particles were harvested and were filtered through Millex‐GP Filter Unit (0.45 µm pore size, Millipore). Liver cancer cells were infected with lentivirus twice, together with 20% FBS and 5 mg mL^−1^ polybrene (Sigma) at 37 °C. To increase the infection efficiency, cells were under 5–7 d of puromycin selection. Purified AAV were purchased from OBiO.

### Western Blot, Coimmunoprecipitation, Ubiquitination Assay, and Immunofluorescence

Cells were collected in lysis buffer (50 × 10^−3^ m Tris–HCl(pH7.5), 0.1%Triton‐100, 1 × 10^−3^ m EDTA, 150 × 10^−3^ m NaCl, 0.1% Nonidet P‐40, protease inhibitor cocktail and phosphatase inhibitor (Selleck)) and lysed at 4 °C by sonication. Proteins were run on 8–12% SDS‐PAGE gels and transferred onto PVDF membranes by wet electrophoretic transfer.

For coimmunoprecipitation, cell lysates (500 µL) were incubated with antibodies or control IgG ((Sigma, I5006) overnight at 4 °C. ProteinA/G agarose beads (50 µL, SantaCruz, sc‐2001) were added to each sample. After 3 h, the beads were washed five times with NP‐40 buffer, followed by western blot. Immunoprecipitated proteins and phosphorylated peptides/residues were performed by Shanghai Applied Protein Technology Co., Ltd.

For the ubiquitination assay, 293T cells were cotransfected with the indicated plasmids, Nickel‐nitrilotriacetic acid (Ni‐NTA) agarose (Qiagen, Inc.) was used to pull down poly‐ubiquitinated HMGCS1. Detailed procedures were performed as previously described.^[^ ^17a^ ^]^

Immunofluorescence staining was performed as previously described.^[^ ^16^ ^]^

### Proteomics Assay

Huh‐7 cells were infected doxycycline inducible shCSN6‐58 (target 3′UTR of CSN6), and then infected with plvx‐CSN6 overexpressing lentivirus. Huh‐7 control group, Huh‐7 shCSN6 group and Huh‐7 CSN6 rescue group were send for proteomics assay by Shanghai Applied Protein Technology Co., Ltd. Immunoprecipitated proteins and phosphorylated peptides/residues were performed by Shanghai Applied Protein Technology Co., Ltd.

### Quantitative Real‐Time PCR (qPCR)

Total RNA was extracted using TRIzol (Thermo Fisher, #15596018) reagent and contaminant DNA was removed by DNase treatment. Retrotranscription was performed and specific gene expression was quantified using SuperReal PreMix SYBR Green (biotool, #B21203) on a lightCycler480 PCR system (Roche). All genes were normalized to β‐actin.

### HE and Immunohistochemistry (IHC)

Human HCC and adjacent matched nontumor tissue samples were obtained from Sun Yat‐sen University Cancer center in Guangzhou, China. The use of human HCC samples was approved by the Sun Yat‐sen University Cancer center Research Ethics Committee and complied with all relevant ethical regulations. The expressions of CSN6, HMGC1, SPOP, YAP, Cleaved‐Caspase 3 and Ki‐67 in tumors were characterized by immunohistochemistry using specific antibodies. In brief, tumor sections (4 µm) were dewaxed in xylene, hydrated in descending concentrations of ethanol, immersed in 0.3% H_2_O_2_‐methanol for 30 min, washed with phosphate‐buffered saline, and probed with monoclonal anti‐CSN6 (1:400), anti‐HMGCS1 (1:200), anti‐SPOP (1:100), anti‐YAP1 (1:200), anti‐Cleaved‐Caspase 3 (1:100) or Ki‐67 antibodies (1:100) or isotype control at 4 °C overnight. After washing, the sections were incubated with biotinylated goat anti‐rabbit or anti‐mouse IgG at room temperature for 2 h. Immunostaining was visualized with streptavidin/peroxidase complex and diaminobenzidine, and sections were then counterstained with hematoxylin.

### Mice

This study was approved by the Animal Ethical and Welfare Committee of the Sixth Affiliated Hospital, Sun Yat‐sen University (20181114‐002). All the mice, including *Csn6* ^fl/fl^ mice and *Alb‐Cre* mice were obtained from Nanjing Biomedical Research Institute of Nanjing University (NBRI). *Csn6* ^fl/fl^ mice were established via CRISPR/Cas9 system and crossed with Alb‐Cre mice. All mice were maintained on a C57BL/6 genetic background. Genotyping and related experiments were performed as previously described.^[^ ^26^ ^]^ To induce HCC, 15 d old mice were injected with 25 mg kg^−1^ DEN (intraperitoneal injection), and tumors were collected around 54 weeks. For acute DEN treatment, 8–12 week old mice were injected with 100 mg kg^−1^ DEN.

For xenograft mice model, the in vivo tumor growth of Huh‐7 cells transduced with shCSN6‐58 or shCSN6‐61 was determined using a subcutaneous transplant xenograft model. Huh‐7 (5 × 10^6^ cells per mouse) cells were inoculated subcutaneously into the hind‐flanks of 5 week old female BALB/c‐nu/nu mice. After 6 d, palpable tumors had developed (≈80 mm^3^), and mice were divided into two groups at random. After 8 d, tumors reached an average size of ≈150 mm^3^. Tumor length and width were measured, and the volume was calculated according to the formula (length × width^2^) /2.

For hydrodynamic tail vein injection (HDTI), HA‐myr‐AKT, N90‐β‐catenin, sleeping beauty (SB), were injected in 2 mL PBS within 7 s.

For orthotropic mice model, Huh‐7 cells were collected in PBS and intrahepatically injected into 6 week old female BALB/c nude mice or C57 mice.^[^ ^27^ ^]^ The liver of each mouse was dissected and then fixed in 4% formaldehyde and embedded in paraffin. Mice were fed with control diet (CD, 18%fat, 58%carbohydrate, 24% protein, 0% cholesterol) or high fat diet (40% fat, 36% carbohydrate, 20% protein, 2% cholesterol) for at list 4 months.

For the patient derived xenograft (PDX), patient‐derived tumor fragments (3–4 mm^3^) were surgically xenografted under the skin of male NSG mice.^[^ ^17a^ ^]^ When tumors reached approximately 100 mm^3^, mice were assigned randomly into four treatment groups.

### Serum Cholesterol, TG, ALT, AST, LDH and Cytokines Measurement

Mouse blood was collected by retro‐orbital bleeding. ALT, AST and LDH, total cholesterol and triglyceride levels were determined by the total cholesterol kit, triglyceride kit and related kit according to the manufacturer's instructions (Servicebio). For cytokines assay, mouse serums were collected and subject to Wayen Biotechnologies (shanghai) for cytokine screening using Bio‐plex Pro Mouse Cytokine Grp 1 Panel 23‐plex (Biorad, M60009RDPD/64377234).

### Luciferase Assays

Luciferase assays were performed in Huh‐7 and SNU‐182 cells with established YAP responsive reporter 8XGTII‐lux (Addgene) and CMV‐Renilla to normalize for transfection efficiency. Cell lysates were analyzed using the Dual‐luciferase Reporter Assay System (Promega, E1910).

### Statistics

All statistical analyses were performed using SPSS 16.0 and/or GraphPad Prism software. Kaplan‐Meier survival analyses were used to compare survival among HCC patients based on CSN6 and HMGCS1 expression; the log‐rank test was used to generate *p* values. Significance was defined as *P* < 0.05. Differences between groups were evaluated using a two‐tailed t test or a Mann‐Whitney rank‐sum test. Paired samples were compared using a paired t test.

## Conflict of Interest

The authors declare no conflict of interest.

## Author Contributions

K.L., J.Z., H.L., and J.Y. contributed equally to this work. K.L., J.Y.Z., B.F.Q., H.W.L., J.N.Y., W.X.W., Y.Z.W., H.D.L., Y.J.Z., X.J., H.R.Y., M.A.W., and H.X. carried out the overall experiments and analyzed the data. Z.G.Z., L.S.X., G.L.L., Y.L.W., F.Y., and R.X.Y. provided the clinical samples and helped analysis of the results. B.F.Q., X.H.F., C.Y.Z., and K.W. performed IHC staining. W.X.W. and H.P.Z. assisted with the in vivo related experiments. K.L., B.F.Q., and W.J.W. established the PDX models. M.H.L., K.L., and H.P.Z. conceived the project, directed the research and co‐wrote the paper.

## Supporting information

Supporting Information

## Data Availability

The data that support the findings of this study are available on request from the corresponding author. The data are not publicly available due to privacy or ethical restrictions.
