# Supplementary material for: CSN6‐SPOP‐HMGCS1 Axis Promotes Hepatocellular Carcinoma Progression via YAP1 Activation
Source: Adv Sci (Weinh). 2024 Feb 2;11(14):2306827. doi: 10.1002/advs.202306827 (PMC11005689; doi:10.1002/advs.202306827)
Supplement: Supplementary file 1 — Supporting Information [file ADVS-11-2306827-s001.pdf]

ADVANCED SCIENCE
Open Access

Supporting Information

for *Adv. Sci.*, DOI 10.1002/advs.202306827

CSN6-SPOP-HMGCS1 Axis Promotes Hepatocellular Carcinoma Progression via YAP1 Activation

Kai Li, Jiayu Zhang, Haiwen Lyu, Jinneng Yang, Wenxia Wei, Yuzhi Wang, Haidan Luo, Yijing Zhang, Xin Jiang, Hairong Yi, Mengan Wang, Caiyun Zhang, Kang Wu, Lishi Xiao, Weijie Wen, Hui Xu, Guolin Li, Yunle Wan, Fang Yang, Runxiang Yang, Xinhui Fu, Baifu Qin, Zhongguo Zhou, Haipeng Zhang* and Mong-Hong Lee**

Supporting Information

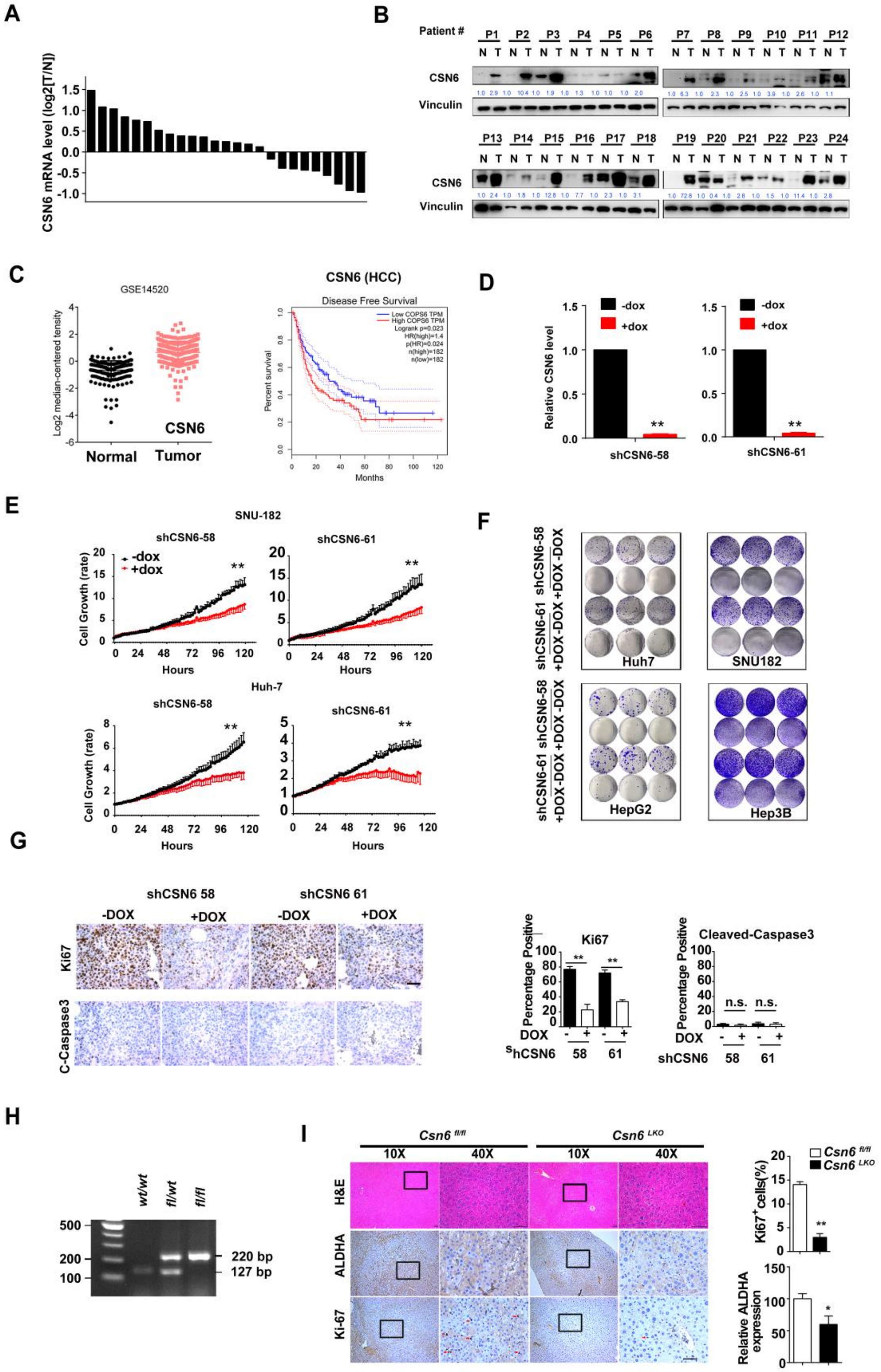

Supplemental Figure 1 CSN6 depletion supresses HCC tumor cell growth.

A Waterfall plot of the CSN6 mRNA levels from paired samples of HCC and adjacent normal tissue as measured by qPCR.

B Expression of CSN6 was detected by western blot in liver cancer and adjacent normal tissue.

C Relative expression of CSN6 in normal and HCC tissue samples from database GSE14520. Kaplan-Meier survival curves of cancer patients from the database (Gepia). High expression of CSN6 is correlated with poor survival in HCC.

D-E Huh-7 cells and SNU-182 cells were infected with the indicated doxycycline (DOX)-inducible shRNAs. qPCR of gene expression in cells with CSN6 knockdown (KD) was shown. Cell proliferation rates were measured. The data are presented as the means ± s.d.. DOX, 100ng/ml. n=3. **, p<0.01.

F Colony formation was measured after DOX-induced CSN6 KD in indicated cells. n=3.

G Representative IHC images of Ki-67 and Cleaved-Caspase 3 in tumor tissues of DOX-induced shCSN6 xenograft tumors (left). Quantification of indicated staining was shown (right). Scale bar, 50 μm. Signals were quantitated and presented as a bar graph. The data are presented as the means ± s.d. n=3. **, p<0.01.

H Genotyping of the *Csn6*$^{fl/fl}$ and *Csn6*$^{fl/fl}$; *Alb-Cre* (*Csn6*^{LKO}) mice. Genotyping of wild type, heterozygous and homozygous mouse were shown.

I Expression of ALDHA and Ki-67 in liver tumor tissues (IHC) from indicated mice after DEN treatment (48h). Signals of ALDHA and Ki-67 were quantitated. Scale bar, 50μm. n=3. **, p<0.01.

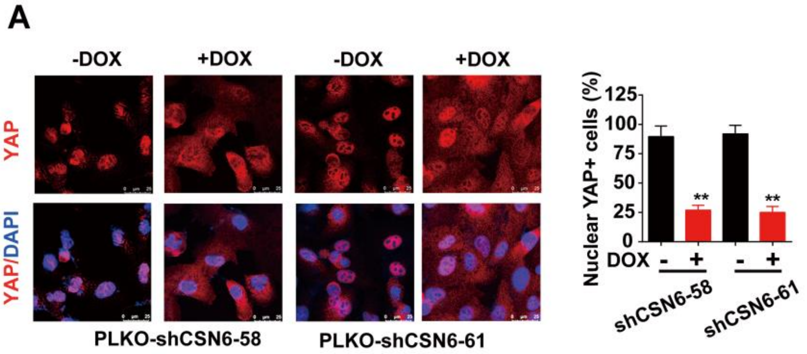

Supplemental Figure 2 CSN6 KD represses YAP1 nuclear translocation.

A Immunofluorescence assay shows silencing CSN6 increased YAP1 translocation from the nucleus to the cytoplasm. n=3. **, p<0.01.

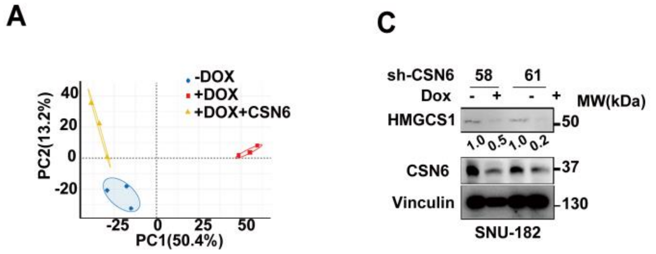

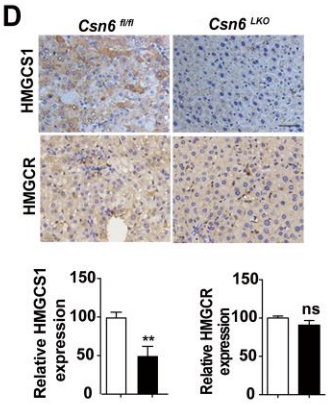

B

Rank	Gene Symbol	Fold change	Rank	Gene Symbol	Fold change
1	COPS6	3.26	36	TLN1	1.76
2	IPO4	2.78	37	CFL1	1.76
3	HMGCS1	2.71	38	NQO1	1.74
4	RANBP1	2.65	39	ENO1	1.73
5	TUBA1C	2.56	40	STAT1	1.72
6	NAP1L1	2.45	41	PSMD11	1.71
7	GPS1	2.43	42	PSMA5	1.70
8	PRMT5	2.38	43	UGDH	1.70
9	EEF2	2.30	44	PSMD13	1.69
10	AARS	2.26	45	STRN3	1.68
11	FASN	2.20	46	DYNC1H1	1.68
12	SLK	2.15	47	LDHA	1.65
13	EEF1A1	2.14	48	CUL2	1.62
14	TUBB	2.09	49	SERBP1	1.61
15	ALDOA	2.06	50	UBL4A	1.61
16	FDPS	2.00	51	ADRM1	1.61
17	COPS5	1.99	52	FLNA	1.61
18	PKM	1.97	53	EIF3B	1.60
19	GAPDH	1.94	54	MYH9	1.57
20	TUBB4B	1.92	55	PSMD12	1.56
21	DYNC1LI1	1.92	56	PSMA1	1.55
22	BAG3	1.90	57	CDK1	1.53
23	HSP90AA1	1.90	58	TAX1BP1	1.53
24	HSPH1	1.89	59	PSMC6	1.53
25	YWHAZ	1.89	60	PSMA4	1.53
26	TXN	1.89	61	PSMD14	1.50
27	GET4	1.88	62	DSP	0.67
28	PTGR1	1.87	63	APLP2	0.60
29	COPS4	1.86	64	SMPD4	0.60
30	SQSTM1	1.85	65	KDM2A	0.59
31	HSP90AB1	1.85	66	TOP2A	0.59
32	WDR77	1.81	67	VSNL1	0.47
33	GRIPAP1	1.79	68	HSPG2	0.46
34	MAPK1	1.78	69	CTSL	0.37
35	MYL12A	1.77			

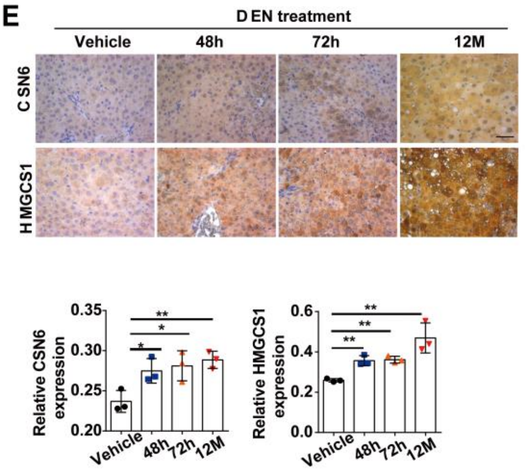

Supplemental Figure 3 HMGCS1 overexpression is correlated with poor clinical outcome of HCC.

A Principal component analysis of proteomics data from control group, CSN6 KD group and CSN6 rescue group. PC1 and PC2 explain 50.4% and 13.2% of the variation, respectively.

B 68 candidate proteins were identified through proteomics.

C Immunoblot of HMGCS1 expression after DOX-induced KD of CSN6.

D Immunohistochemistry analysis of HMGCS1 and HMGCR in liver tissue of DEN/CCl_4-treated *Csn6*$^{fl/fl}$ and *Csn6*^{LKO} mice. Scale bar, 50μm. n=3. **, p<0.01. ns, not significant.

E Tissues were evaluated through IHC for CSN6 and HMGCS1 expression from DEN/CCl_4-treated mice. Quantitative results were shown. Scale bars, 50 μM. n=3. **, p<0.01.

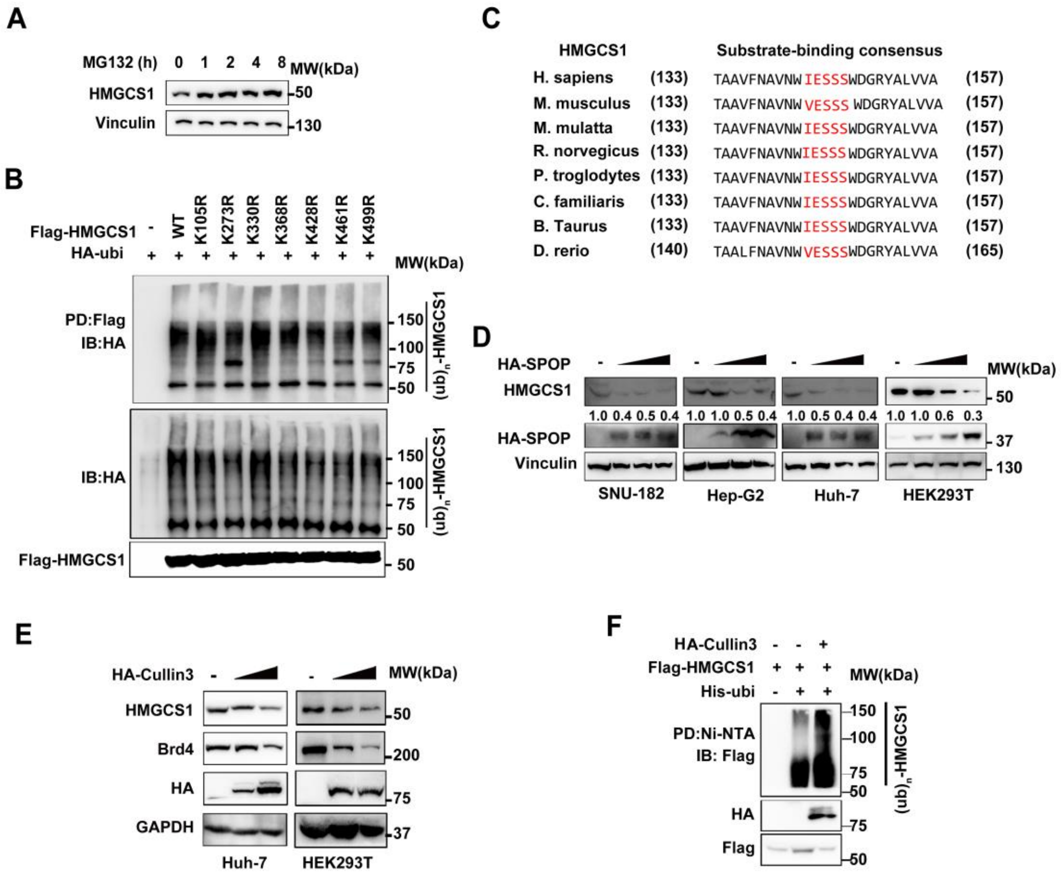

Supplemental Figure 4 CSN6 mediated Cul3-SPOP complex blockade is involved in HMGCS1 dysregulation.

A Steady-state expression of HMGCS1 protein is regulated by proteasome. MG132 stabilized HMGCS1 in 293T cells.

B Single mutation of each lysine residue on HMGCS1 is still vulnerable to ubiquitination. Cells transfected with indicated constructs were treated with MG132 (10 μM) 6 h before harvest. The cell lysates were pulled down (PD) with M2 beads and immunoblotted with indicated antibodies.

C Amino acid sequence alignment of putative SPOP binding consensus (SBC) motifs in HMGCS1.

D Representative immunoblots showing HMGCS1 steady-state expression in indicated cells upon SPOP overexpression. RT-PCR analysis of HMGCS1 in 293T cells transfected with SPOP. SPOP overexpression does not affect HMGCS1 mRNA expression.

E Representative immunoblots showing HMGCS1 steady-state expression in indicated cancer cells transfected with Cullin3. Cullin3 overexpression decreased HMGCS1 steady-state expression in Huh-7 and 293T cell line.

F Cullin3 overexpression increased HMGCS1 poly-ubiquitination in 293T cells.

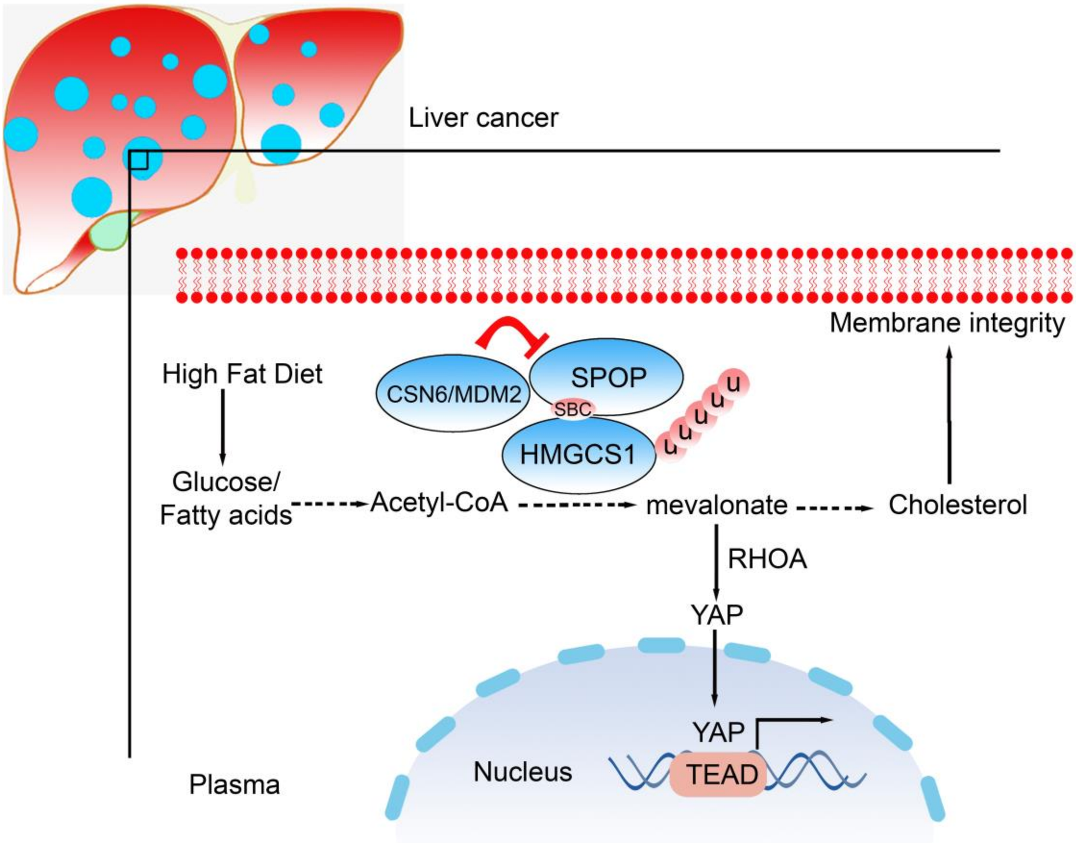

Supplemental Figure 5 A simplified model depicting the regulatory role of CSN6 and SPOP in mevalonate metabolism and HCC development. Mevalonate activates YAP is mediated by Rho GTPase (RHOA). TEAD is a well-known YAP co-transcriptional factor for target genes expression.
